# Ultra-high field MRI reveals mood-related circuit disturbances in depression: a comparison between 3-Tesla and 7-Tesla

**DOI:** 10.1038/s41398-019-0425-6

**Published:** 2019-02-15

**Authors:** Laurel S. Morris, Prantik Kundu, Sara Costi, Abigail Collins, Molly Schneider, Gaurav Verma, Priti Balchandani, James W. Murrough

**Affiliations:** 1 The Mood and Anxiety Disorders Program, Department of Psychiatry, The Icahn School of Medicine at Mount Sinai, New York, NY 10029 USA; 2 The Translational and Molecular Imaging Institute, Department of Radiology, The Icahn School of Medicine at Mount Sinai, New York, NY 10029 USA

## Abstract

Ultra-high field 7-Tesla (7 T) MRI has the potential to advance our understanding of neuropsychiatric disorders, including major depressive disorder (MDD). To date, few studies have quantified the advantage of resting state functional MRI (fMRI) at 7 T compared to 3-Tesla (3 T). We conducted a series of experiments that demonstrate the improvement in temporal signal-to-noise ratio (TSNR) of a multi-echo multi-band fMRI protocol with ultra-high field 7 T MRI, compared to a similar protocol using 3 T MRI in healthy controls (HC). We also directly tested the enhancement in ultra-high field 7 T fMRI signal power by examining the ventral tegmental area (VTA), a small midbrain structure that is critical to the expected neuropathology of MDD but difficult to discern with standard 3 T MRI. We demonstrate up to 300% improvement in TSNR and resting state functional connectivity coefficients provided by ultra-high field 7 T fMRI compared to 3 T, indicating enhanced power for detection of functional neural architecture. A multi-echo based acquisition protocol and signal denoising pipeline afforded greater gain in signal power compared to classic acquisition and denoising pipelines. Furthermore, ultra-high field fMRI revealed mood-related neurocircuit disturbances in patients with MDD compared to HC, which were not detectable with 3 T fMRI. Ultra-high field 7 T fMRI may provide an effective tool for studying functional neural architecture relevant to MDD and other neuropsychiatric disorders.

## Introduction

In the past 30 years, functional magnetic resonance imaging (MRI) has provided unprecedented insight into the neural mechanisms of neuropsychiatric disorders in humans, including major depressive disorder (MDD). Work with 3-Tesla (3 T) functional MRI has revealed the functional architecture of key neural systems that contribute to the neuropathology of MDD, including aberrant connectivity of limbic and reward-related networks that subserve mood regulation^1–4^. The next generation of ultra-high field 7-Tesla (7 T) MRI magnets has just been approved by the US Food and Drug Administration (FDA) for clinical use and while it has significant potential to advance our understanding of neuropsychiatric disorders, there remain concerns about data quality and systematic comparisons between 7 and 3 T functional MRI remain scarce^5–8^.

Ultra-high field 7 T MRI benefits from increased signal to noise ratio (SNR)^5,7^, enhanced amplitude and percent of signal change^5,8,9^, and increased susceptibility induced and blood oxygen level dependent (BOLD) contrast, all important for functional and spectroscopic applications^6^. However, higher field strengths also produce more B0 inhomogeneities and susceptibility artifacts, which lead to geometric distortion, signal dropout and signal pile-up^6^. These distortions are most severe around tissue-air boundaries and more subtle susceptibility distortions can arise from paranasal sinuses and bone^10^. Ventral portions of the brain, including subcortical and midbrain structures important for mood regulation, are particularly affected by susceptibility distortions and signal drop-out due to their proximity to bone and sinuses^11,12^. These limitations have led to concerns regarding the utility of ultra-high field MRI for examining ventral regions relevant to neuropsychiatric disorders. Shorter echo-time (TE), thinner slices and parallel imaging can combat some of these issues by reducing intra-voxel inhomogeneity and through-plane dephasing^6,9,13^. We have developed and implemented an ultra-high field 7 T functional MRI scanning protocol with multiple TE’s, thin slices, multi-band acquisition and TE-dependent physiological denoising methods to improve signal power detection and temporal SNR (TSNR) for in vivo imaging.

The application of ultra-high field 7 T functional MRI to the study of neuropsychiatric disorders such as MDD remains scarce. MDD is one of the world’s largest public health issues to date, affecting approximately 300 million people and representing the leading cause of disability worldwide^14^. The current lack of widely effective treatments demonstrates in part our limited understanding of the etiology and biological mechanisms of this complex disorder. One of the core symptoms of MDD is anhedonia, a markedly diminished response to pleasure, which is characterized by underlying dysfunction of limbic and reward-related neural systems centered on VTA, nucleus accumbens and anterior cingulate cortex (ACC)^15–17^. VTA hyperactivity and hyperconnectivity has been demonstrated in a well-validated preclinical model of depression that is characterized by anhedonic and other pro-depressive behaviors^18–20^. VTA connectivity, however, has not been fully explored in humans with MDD, partly due to limited feasibility of discerning VTA at standard 3 T field strength^11,12^.

Here, we detail a series of experiments that demonstrate the functional MRI signal quality of a multi-echo scanning protocol at ultra-high field 7 T MRI, and compare it to 3 T MRI. Our first aim was to compute the gain in TSNR with ultra-high field 7 T MRI, both across the whole brain and in particular neural regions relevant to MDD and related neuropsychiatric disorders. We subsequently examined functional connectivity of three essential fronto-striatal-midbrain circuits that mediate cognitive, limbic and motor functions^21–23^ using ultra-high field 7 T as compared to 3 T MRI. Our second aim was to characterize the expected improvement in signal power more directly in healthy controls (HC) and patients with MDD by examining the functional connectivity of the VTA. We specifically selected the VTA due to its critical role in the expected neuropathology of MDD coupled with the difficulty in its delineation with 3 T MRI. We hypothesized that whole-brain TSNR and circuit-specific functional connectivity would be improved at ultra-high field 7 T compared to 3 T, and further that 7 T functional MRI would reveal VTA circuit differences between MDD and HC.

## Methods

### Participants

All subjects were recruited at the Mood and Anxiety Disorder Program (MAP) at Icahn School of Medicine at Mount Sinai. All eligible participants between the ages of 18–65 underwent the Structured Clinical Interview for DSM-V Axis Disorders (SCID-V) by a trained rater to determine any current or lifetime psychiatric disorder^24^. Subjects were excluded if they had an unstable medical illness, history of neurological disease, history of schizophrenia or other psychotic disorder, neurodevelopmental/neurocognitive disorder, substance use disorder (SUD) within the past 2 years, any contraindications to MRI, or positive urine toxicology on the day of scan. HC subjects were free from any current or lifetime psychiatric disorder. All participants were free of antidepressant medication or other psychotropic medication for at least 4 weeks (8 weeks for fluoxetine) prior to data collection. Inclusion criteria for MDD subjects included having MDD as their primary presenting problem and being in a current major depressive episode (MDE). In all subjects, depressive symptom severity was measured with the Montgomery–Åsberg Depression Rating Scale (MADRS)^25^ and severity of anhedonia was measured with the Snaith-Hamilton Pleasure Scale (SHAPS)^26^. Subjects underwent either 3 or 7 T MRI and a subset of individuals completed both 3 and 7 T. All data was collected under Institutional Review Board (IRB)—approved written informed consent. All subjects were compensated for their time.

### MRI acquisition and preprocessing

Functional MRI data was collected with 3 T Siemens Magnetom Skyra and 7 T Siemens Magnetom scanners, with 32-channel head coil, using a 10-min multi-echo multi-band echo-planar imaging (EPI) pulse sequence with the following parameters at 3 T: 3 mm isotropic resolution, 45 slices, TR/TE’s = 882/11, 29.7, 48.4, 67.1 ms, MB = 5, iPAT acceleration factor = 2664 frames, flip = 45, field of view = 560 × 560, pixel bandwidth = 2085; and at 7 T: 2.5 mm isotropic resolution, 50 slices, TR/TE’s = 1850/8.5, 23.17, 37.84, 52.51, MB = 2, iPAT acceleration factor = 3, 300 frames, flip = 70, field of view = 640 × 640, pixel bandwidth = 1786. Anatomical data were collected at 3 T and at 7 T with a twice magnetization-prepared rapid gradient echo (MP2RAGE) sequence for improved T1-weighted contrast and spatial resolution^27,28^, with the following parameters at 3 T: 1 mm isotropic resolution, 56 slices, TR/TE = 4000/1.9 ms, field of view = 184 × 160, bandwidth = 250; and at 7 T: 0.7 mm isotropic resolution, 60 slices, TR/TE = 6000/3.62 ms, field of view = 240 × 320, bandwidth = 300.

Functional data were processed and denoised for motion and physiological artefacts using freely available multi-echo independent components analysis (ME-ICA, https://bitbucket.org/prantikk/me-ica)^29^. This is explained in detail elsewhere^29,30^. Briefly, the ME-ICA package exploits the property that BOLD percent signal change is linearly dependent on TE, a consequence of T2* decay^29,30^. ME-ICA decomposes multi-echo functional MRI data into independent components, and computes the TE dependence of the BOLD signal for each component, as measured using the pseudo-*F*-statistic, Kappa, with components that scale strongly with TE having high Kappa scores^29^. Non-BOLD components are identified by TE independence measured by the pseudo-*F*-statistic, Rho. As such, components are categorized as BOLD or non-BOLD based on their weightings measured by Kappa and Rho values, respectively. By removing non-BOLD components, data are denoised for motion, physiological and scanner artefacts in a robust manner based on physical principles^29,30^. Denoised functional data were coregistered with T1 and normalized to a standard template with Advance Normalization Tools (ANTs, http://stnava.github.io/ANTs/) software using diffeomorphic symmetric normalization transformation (SyN) registration^31,32^.

Data from TE2 (29.7 ms at 3 T and 23.17 at 7 T) were processed using a standard denoising pipeline implemented in AFNI software (afni_proc.py^33^), which included whole brain voxel timeseries despiking, correction for slice timing offsets, image realignment to a middle slice for volume-to-volume rigid body correction, masking, regression of demeaned motion parameters plus derivatives and censorship of time points with motion >0.2 mm, and spatial smoothing with a full width half maximum kernel of 6 mm (these procedures including smoothing were performed for the single-echo data only). While this does not represent an optimal single-echo functional MRI protocol, it provides a useful illustrative comparison.

### Data analysis

Demographic data and clinical characteristics were summarized and compared using independent *t*-tests, including for the clinician-administrated scale of depression severity (MADRS) and the self-report measure of anhedonia (SHAPS) (Table 1).

**Table 1:** Demographic and clinical characteristics

	3 T		7 T	
	MDD	HC	MDD	HC
*N*	15	17	10	17
Age at enrollment, years (mean ± SD)	40.7 ± 11.560	37.4 ± 10.137	34.3 ± 11.186	38.41 ± 11.9
Male (frequency, %)	9, 60%	12, 70.6%	6, 60%	9, 52.9%
White (frequency, %)	8, 53.3%	8, 47.1%	6, 60%	6, 46.2%
Hispanic ethnicity (frequency, %)	6, 40%	1, 5.9%	2, 20%	2, 15.4%
College degree, at least 2-year (frequency, %)	12, 80%	15, 88.3%	8, 80%	10, 76.9%
Employed, at least part-time (frequency, %)	10, 66.7%	14, 82.4%	7, 70%	11, 84.6%
Married (frequency, %)	1, 6.7%	3, 17.6%	0%	2, 15.4%
Age at first episode (mean ± SD)	23.6 ± 13.695	–	22 ± 12.055	–
Years since first episode (mean ± SD)	17.1 ± 10.575	–	12.3 ± 6.913	–
Number of episodes (median, range)	1, 39	–	2.5, 48	–
Duration of current MDE, months (median, range)	120, 498	–	13.5, 190	–
Recurrent MDD (frequency, %)	4, 26.7%	–	6, 60%	–
SHAPS Score (mean ± SD)	35.27 ± 7.206	17.1 ± 4.293	39.6 ± 5.966	18.7 ± 4.9
MADRS score (mean ± SD)	28.4 ± 4.687	0.824 ± 1.131	29.8 ± 7.569	0.5 ± 0.9

Six of the 17 healthy control (HC) and 5 of the major depressive disorder (MDD) subjects completed both 3-Tesla (3 T) and 7-Tesla (7 T)

*N* number of subjects, *MADRS* Montgomery–Åsberg Depression Rating Scale, *SHAPS* Snaith-Hamilton Pleasure Scale, *SD* standard deviation

### Experiment 1

For both 3 and 7 T functional MRI data, temporal SNR (TSNR) was computed as the mean/standard deviation of the voxel signal timecourses of the raw EPI data for each TE and of the denoised data before coregistration and normalization across the whole brain. Using the functional MRI data normalized to template space, TSNR was also computed for the 7 regions that subserve cognitive and behavioral processes relevant to psychiatric research: ventral tegmental area, nucleus accumbens, amygdala, subgenual ACC, dorsal ACC, ventromedial prefrontal cortex (PFC), and dorsolateral PFC. The anatomical boundaries and definitions of these regions are described in detail elsewhere^34^.

With the normalized functional MRI data at 3 and 7 T, a 7 × 7 cross-correlation matrix was computed for the seven regions of interest using standard Pearson correlation of the mean timecourse of each region, followed by Fisher’s R-to-Z transformation of the correlation coefficient^35^. Percent differences were computed between 3 and 7 T Fisher Z-transformed correlation coefficients.

In addition, seed-to-voxel functional connectivity maps were computed for the VTA, ventromedial PFC, dorsolateral PFC and SMA to examine networks relevant to depression as well as three cortical-basal ganglia circuits relevant to broader psychiatric research^23,34^. For seed-to-voxel functional connectivity maps, whole-brain statistical cluster-correction was performed with AFNI’s 3dClustSim, correcting for the instance of false positives due to multiple comparisons and spatial autocorrelation^36^. The correction threshold for the 3 T data was calculated at voxelwise *p* < 0.001 and Cluster > 27 voxels for alpha < 0.05. At 7 T it was at voxelwise *p* < 0.001 and Cluster > 919 voxels for alpha < 0.05.

### Experiment 2

For the examination of patients compared to controls, we primarily examined seed-to-seed functional connectivity, computed as the Pearson correlation between averaged timecourses for VTA and nucleus accumbens; VTA and subgenual ACC; and VTA and dorsal ACC. These were entered into independent samples *t*-test to compare between groups at both 3 and 7 T. For this *p* < 0.017 was considered significant, with bonferonni correction for 3 comparisons. Seed-to-seed functional connectivity for these regions was additionally correlated with duration of current episode and the depressive symptom of anhedonia.

On an exploratory basis, VTA seed-to-whole-brain functional connectivity maps were compared at both 3 and 7 T using independent samples *t*-test. For this exploratory whole-brain analysis, we examined the data using the following threshold: for 3 T, voxelwise *p* < 0.01 and Cluster > 104 for alpha < 0.05; and for 7 T, voxelwise *p* < 0.01 and Cluster > 4645 for alpha < 0.05.

## Results

### Experiment 1

#### Signal power at high field MRI in healthy controls

Seventeen healthy control (HC) participants were recruited and scanned on 3 T (5 female, age = 37.4 ± 10.1) and 17 on 7 T (8 female, age = 38.41 ± 11.9). 6 of the 17 subjects completed both 3 and 7 T scans (1 female, age = 36.8 ± 11.6). See Table 1 for clinical and demographic details.

#### Shorter and multiple TE’s contribute to improved SNR at high field

**Fig. 1:**
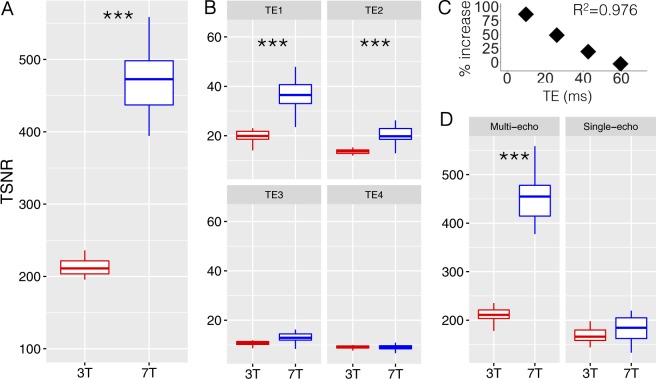
Higher temporal signal to noise ratio (TSNR) with ultra-high field 7-Tesla (7 T) functional magnetic resonance imaging (MRI) scales with echo time (TE) and is boosted by multi-echo based denoising. **a** TSNR plotted for whole brain at 3-Tesla (3 T) and 7 T. **b** TSNR plotted for 7 T at 3 T for 4 different TE’s (TE1 = shortest, TE4 = longest). **c** Percent increase in TSNR at 7 T compared to 3 T scales with TE (Linear fit, adjusted *R*^2^ = 0.976, *p* = 0.008). **d** TSNR plotted for whole brain after multi-echo acquisition and denoising and traditional single-echo acquisition and denoising. ***p ≤ 0.001

Whole brain multi-echo denoised EPI data had 127.98% higher TSNR at 7 T compared to 3 T (7 T TSNR: 475.3 ± 85.2 (18% SD); 3 T TSNR: 214.7 ± 41.6 (19% SD); *t*_(32)_ = 11.6, *p* = 2.7 × 10^−12^; Fig. 1a). Raw EPI TSNR was also examined for each TE. Whole brain TSNR was increased at 7 T compared to 3 T at shorter TEs but not longer TEs (TE1 = 76.29%, *t*_(32)_ = 5.5, *p* = 3.6 × 10^−5^; TE2 = 40.96%, *t*_(32)_ = 4.0, *p* = 0.001; TE3 = 12.87%, *t*_(32)_ = 1.8, *p* = 0.096; TE4 = −7.7%, *t*_(32)_ = −1.6, *p* = 0.144; Fig. 1b). The percent improvement in TSNR from 3 to 7 T was linearly related to TE (adjusted *R*^2^ = 0.98, *p* = 0.008, Fig. 1c).

Multi-echo EPI data acquisition and denoising methods improved TSNR compared to conventional single echo acquisition and denoising (afni_proc.py with TE2) by 48.4% at 7 T (*t*_(16)_ = 15.7, *p* = 3.8 × 10^−11^) but not at 3 T (Fig. 1d), indicating increased SNR gain produced by ME-ICA at 7 T compared to 3 T. Motion was not significantly different between 3 and 7 T (millimeter displacement, *p* = 0.37; RPY degrees, *p* = 0.68).

The same pattern of improved TSNR at 7 T compared to 3 T for whole brain denoised EPI and native individual TE EPI data was observed when comparing 5 MDD subjects who completed 3 and 7 T (whole-brain TSNR at 3 T = 211.6 ± 25.1; 7 T = 425.3 ± 42.5; *p* = 0.006) and 5 HC subjects who completed both 3 and 7 T (whole-brain TSNR at 3 T = 200.1 ± 52.6; 7 T = 507.8 ± 110.2; *p* = 0.005; Supp Fig. 1).

#### Higher field strength yields improved SNR in ventral regions relevant to neuropsychiatric disorders

**Fig. 2:**
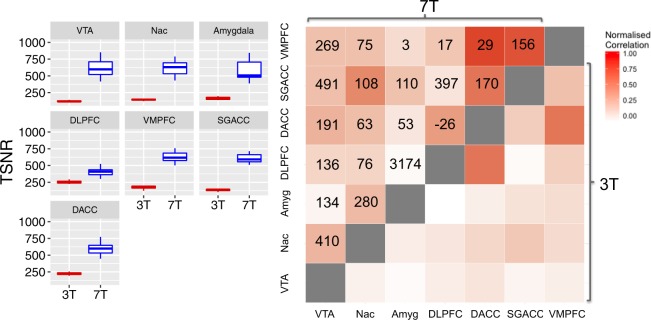
Improvement in ultra-high field 7-Tesla (7 T) functional magnetic resonance imaging (MRI) signal power and cross-correlation coefficients throughout the brain. Left: Temporal signal to noise ratio (TSNR) plotted for regions of interest at 3-Tesla (3 T) and 7 T. Right: Matrix of Fisher Z-transformed cross-correlation coefficients between the same brain regions at 3 T (bottom right) and 7 T (top left). Values indicate percent improvement in correlation coefficient at 7 T compared to 3 T. VTA, Ventral tegmental area; Nac, nucleus accumbens; Amyg, amygdala; DLPFC, dorsolateral prefrontal cortex; VMPFC, ventromedial prefrontal cortex; SGACC, subgenual anterior cingulate cortex; DACC, dorsal anterior cingulate cortex

Regional TSNR was examined for 7 brain regions relevant to psychiatric research. Similar to whole brain improvement in TSNR at 7 T compared to 3 T, all 7 regions showed increased TSNR at higher field, including ventral structures close to sinuses and bone (Fig. 2a, Supp Table 1, Supp Fig. 3). The same pattern of improved TSNR at 7 T compared to 3 T for regional data was observed when comparing 5 HC subjects who completed both 3 and 7 T (Supp Fig. 1).

Correlation coefficients between these 7 brain regions were compared between 3 and 7 T. Normalized coefficients were higher at 7 T compared to 3 T (Fig. 2b). The pattern of connectivity was similar at 3 and 7 T. For example, the subgenual cingulate cortex had the highest correlation coefficient (functional connectivity) with NAc and lowest with dlpfc at both 3 and 7 T (Fig. 2b). The average percent improvement from 3 to 7 T across this diverse cross-correlation matrix was 300.9%. The same pattern was observed in the same HC and MDD subjects scanned on both 3 and 7 T (Supp Table 2). To determine whether 3 and 7 T correlation patterns were statistically similar, we examined normalized cross-correlation coefficients at 3 and 7 T for 470 approximately same sized regions based on the Harvard-Oxford Atlas^37^, finding significant correlation between 3 and 7 T 470 × 470 cross-correlation matrices (*R* = 0.38, *p* < 0.0001).

**Fig. 3:**
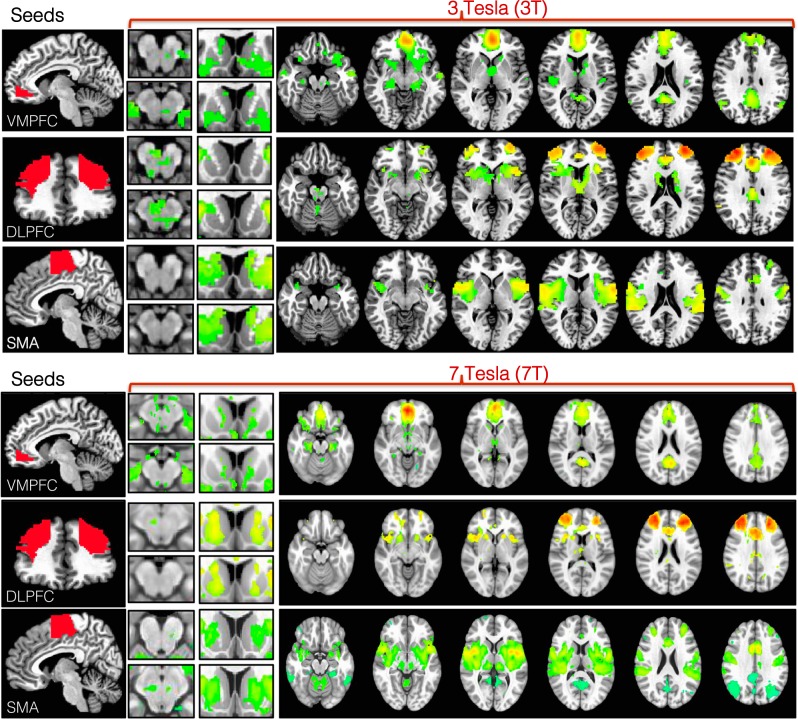
Functional connectivity of cortical-striatal-midbrain circuits with 3-Tesla (3 T) and 7-Tesla (7 T) functional magnetic resonance imaging (MRI). Resting state functional connectivity of three cortical seeds of interest (left, red) including ventromedial prefrontal cortex (VMPFC), dorsolateral prefrontal cortex (DLPFC), and supplementary motor area (SMA) was computed in healthy controls at 3 T (top) and 7 T (bottom). Connectivity maps are thresholded at *p* < 0.0001 voxelwise

Other cortical-basal ganglia circuits relevant to neuropsychiatric research were compared between 3 and 7 T. Three broadly dissociable putative circuits were examined, thought to subserve discrete motor, cognitive and limbic functions, based on SMA, dlpfc and vmpfc connectivity, respectively^23,34^. At 3 T, cortical—basal ganglia circuits were distinguishable, reflecting dissociable motor (SMA, motor cortex, putamen), cognitive (dlpfc, MD thalamus) and limbic (vmpfc, nucleus accumbens, some VTA) subcircuits (Fig. 3). However, at 7 T the precision of identification of each subcircuit was heightened (Fig. 3; Table 2 for statistics). For example, motor circuit nodes SN, posterior STN, ventrolateral thalamus were apparent; cognitive circuit node dorsal caudate; and limbic circuit nodes of VTA, locus coeruleus were apparent (Fig. 3).

**Table 2:** Statistics of connectivity of seed regions of interest with 3-Tesla (3T) and 7-Tesla (7T) functional magnetic resonance imaging

3T							7T						
Region	*Z*	corr	*K*	*x*	*y*	*z*	Region	*Z*	corr	*K*	*x*	*y*	*z*
VMPFC seed							VMPFC seed						
VMPFC	14.3	<0.05	7300	1.5	−50.5	−6	VMPFC	14.3	<0.05	146,283	1	−52	−5
PCC	7.2	<0.05	1241	−1.5	54.5	18	PCC	8.1	<0.05	34,842	4	56	19
Cerebellum	5.6	<0.05	202	−34.5	78.5	−39	OFC	6.1	<0.05	4089	37	−31	−15
Laterial parietal	5.4	<0.05	193	43.5	72.5	39	Cerebellum	5.4	<0.05	3647	32	78	−42
Cerebellum	7.3	<0.05	169	31.5	75.5	−36	Laterial parietal	−5.6	<0.05	3593	29	53	68
Laterial parietal	6.3	<0.05	165	−49.5	60.5	27	Occipital	−5.7	<0.05	2465	10	79	49
Cerebellum	5.9	<0.05	152	−7.5	54.5	−45	Occipital	−6.6	<0.05	1883	−40	81	18
Cerebellum	4.4	<0.05	41	−1.5	57.5	−21	Medial parietal	−5.3	<0.05	1475	−10	68	60
Occipital	4.3	ns	21	25.5	99.5	−12	Laterial parietal	−5.3	<0.05	982	−30	50	44
Temporal	4.1	ns	17	61.5	30.5	−9	Laterial parietal	−4.9	<0.05	946	55	37	43
DLPFC seed							DLPFC seed						
DLPFC	10.9	<0.05	9145	−28.5	−47.5	33	DLPFC	12.1	<0.05	167,874	−31	−50	26
Laterial parietal	6.9	<0.05	278	−55.5	45.5	48	Cerebellum	8.9	<0.05	9252	34	54	−36
Laterial parietal	6.5	<0.05	155	52.5	54.5	48	Cerebellum	6.1	<0.05	5887	−40	52	−37
Cerebellum	4.7	<0.05	74	−19.5	42.5	−45	Laterial parietal	5.8	<0.05	4369	58	48	46
Cerebellum	4.2	<0.05	36	10.5	45.5	−42	Laterial parietal	5.4	<0.05	4284	−57	37	46
Medial parietal	5.4	ns	24	−10.5	69.5	45	Medial OFC	−4.8	<0.05	1170	0	−33	−22
Cerebellum	4.1	ns	22	−13.5	78.5	−27	Medial parietal	9.5	<0.05	958	−16	66	38
Occipital	−4.3	ns	14	−28.5	69.5	−9	Occipital	−4.9	<0.05	942	−16	92	−2
							Medial parietal	4.1	ns	816	12	62	51
							Brainstem	5.5	ns	674	7	44	−60
SMA seed							SMA seed						
SMA	11.9	<0.05	9817	−1.5	9.5	66	SMA	16.3	<0.05	277,708	−1	8	63
Laterial parietal	−5.3	<0.05	140	−37.5	72.5	−48	Laterial parietal	−13.4	<0.05	17,095	−38	71	44
Cerebellum	−6.8	<0.05	116	43.5	66.5	42	PCC	−7.1	<0.05	13,951	−8	46	35
DLPFC	6.7	<0.05	79	31.5	−41.5	30	Laterial parietal	−10.6	<0.05	11,912	33	77	39
Cerebellum	−4.7	<0.05	64	37.5	69.5	−48	Cerebellum	5.8	<0.05	7978	16	45	−25
Cerebellum	5.2	<0.05	43	25.5	54.5	−54	DLPFC	−5.5	<0.05	7850	−31	−9	45
Laterial parietal	−4.7	<0.05	42	−43.5	69.5	42	DLPFC	7.5	<0.05	4739	32	−45	23
Motor Cortex	−6.2	<0.05	40	40.5	−17.5	48	Thalamus	14.3	<0.05	4625	−10	22	2
PCC	−4.1	<0.05	32	−1.5	51.5	36	Thalamus	10.9	<0.05	3803	8	24	2
Thalamus	4.3	<0.05	27	−10.5	18.5	0	DLPFC	−5.9	<0.05	2648	25	−22	50
VTA seed							VTA seed						
VTA	3.3	<0.05	59	−4.5	21.5	−15	VTA	11.1	<0.05	144,215	−2	19	−16
							ACC/mPFC	8.2	<0.05	37,531	−4	−27	16
							DLPFC	4.9	<0.05	1568	27	−48	35
							VLPFC	5.9	ns	528	20	−53	6
							Cerebellum	4.1	ns	505	−33	56	−33
							DLPFC	4.3	ns	378	−27	−30	40
							Anterior insula	4.2	ns	264	−41	−9	9
							Medial parietal	4.2	ns	224	−14	1	51
							Posterior insula	4.2	ns	197	45	21	10
							Medial parietal	4.6	ns	186	12	61	40

*VMPFC* ventromedial prefrontal cortex, *DLPFC* dorsolateral prefrontal cortex, *SMA* supplementary motor area, *VTA* ventral tegmental area, *PCC* posterior cingulate cortex, *OFC* orbitfrontal cortex, *ACC* anterior cingulate cortex, *mPFC* medial prefrontal cortex, *Z Z*-score statistic, *corr* whole-brain cluster corrected significance, *ns* not significant, *K* cluster size, *xyz* peak voxel coordinates

### Experiment 2

#### Reward-related neural network properties at high field in healthy controls and MDD

Subjects meeting DSM-5 criteria for MDD (*N* = 15) were compared to the same HC cohort scanned at 3 T described above (see Table 1 for demographics). A second cohort of patients meeting DSM-5 criteria for MDD (*N* = 10) was compared to the same HC scanned at 7 T described above (see Table 1 for demographics). Five of these MDD subjects completed both 7 and 3 T scans. MDD patients had significantly higher depressive symptoms as measured by the Montgomery–Åsberg Depression Rating Scale (MADRS) and anhedonia as measured by the Snaith-Hamilton Pleasure Scale (SHAPS), compared to HC (*p*’s < 0.001, see Table 1).

#### High field reveals specific neurocircuit disturbances in depression

**Fig. 4:**
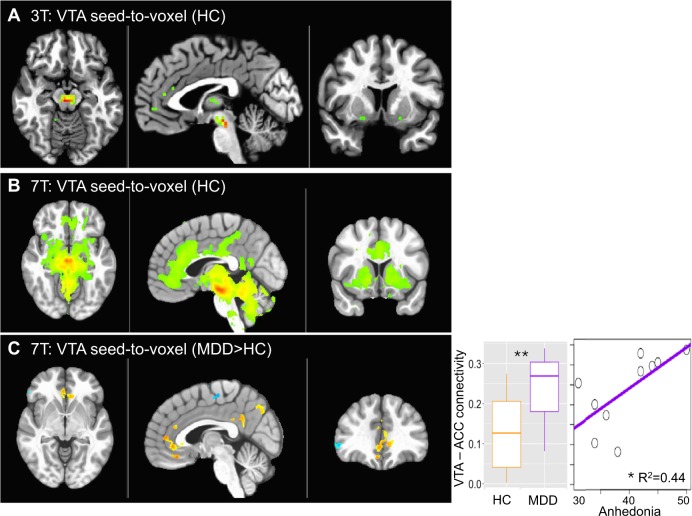
Functional connectivity of the ventral tegmental area (VTA) with 3-Tesla (3 T) and 7-Tesla (7 T) functional magnetic resonance imaging (MRI). Connectivity of the VTA with whole brain is shown for 3 T (**a**) and 7 T (**b**) in healthy controls (HC) (voxelwise *p* < 0.001 for illustration). **c** VTA-to-whole brain functional connectivity comparison between patients with major depressive disorder (MDD) and HC (*p* < 0.01 voxelwise, Cluster > 200). Seed-to-seed VTA-ACC connectivity is plotted for MDD and HC and against anhedonia in the MDD group. **p < 0.01; *p < 0.05

Firstly, VTA seed to voxel connectivity maps were compared at 3 and 7 T in HC only. While at 3 T, there was one significant cluster of VTA connectivity with neighboring midbrain regions, higher field 7 T provided more robust and detailed connectivity maps of the VTA, with medial PFC, ACC, DLPFC at the same whole-brain cluster-corrected thresholds (Fig. 4a, b; Table 1 for statistics).

Given the specific interest in VTA connectivity with NAc and ACC (both subgenual and dorsal divisions) in MDD^18–20^, we examined ROI-to-ROI connectivity between these regions. At 3 T, there were no significant group differences between VTA-NAc or VTA-ACC (subgenual and dorsal) (*p*’s > 0.05). However, at 7 T, MDD subjects showed increased connectivity between VTA-subgenual ACC (*p* = 0.009, Fig. 4c). VTA-subgenual ACC connectivity in the MDD group positively correlated with the duration of the current episode (*R* = 0.70, *p* = 0.035) and the depressive symptom of anhedonia (*R* = 0.67, *p* = 0.036, Fig. 4c). VTA- subgenual ACC connectivity was not correlated with age (*p* > 0.05) or different based on gender (*p* > 0.05) across the sample. There were no group differences in VTA-NAc connectivity or correlations between VTA-NAc connectivity and disorder severity or duration. Importantly, whole brain TSNR did not differ as a function of group between field strengths (Supp Fig. 2).

Whole-brain functional connectivity of the VTA was then compared between MDD patients and HC in an exploratory manner. At 3 T, the comparison between MDD subjects (*N* = 15) and HC (*N* = 17 from Experiment 1) revealed no clusters at voxelwise *p* < 0.01, Cluster > 100. At 7 T, the comparison between a smaller sample of MDD subjects (*N* = 10) and HC (*N* = 17 from Experiment 1) revealed several clusters at voxelwise *p* < 0.01, Cluster > 100, with increased connectivity with ACC in MDD as the top cluster (Fig. 4c; Table 2). While this observation corroborates the ROI-to-ROI based analysis, it did not reach whole brain cluster-corrected significance in this sample (Cluster > 4645 for alpha < 0.05) and should therefore be interpreted with some caution.

## Discussion

We report improved signal power across the brain with ultra-high field 7 T functional MRI compared to 3 T that is boosted by multi-echo based denoising. Functional connectivity coefficients between diverse brain regions relevant to MDD and other neuropsychiatric disorders was significantly enhanced at 7 T compared to 3 T, including of VTA, a small midbrain structure that is limited by poor TSNR at lower clinical field strength. Furthermore, hyperconnectivity between the VTA and subgenual ACC in MDD was revealed at 7 T, consistent with pre-clinical findings in a rodent model of depression^19,20^, while this alteration was not observed at 3 T. Together, this work indicates the considerable utility of ultra-high field 7 T for characterizing pathological alterations in neural architecture relevant to neuropsychiatric disorders.

While clinical applications of ultra-high field 7 T neuroimaging have been posited for multiple disorders including stroke, epilepsy, multiple sclerosis^6^, there has been less emphasis on more subtle neuropsychiatric disorders such as MDD. This work adds to other demonstrations of the utility of ultra-high field functional MRI, including comparisons of Go/No-Go task based functional MRI^38^ and of the resting state connectivity of the habenula^39^. Determining biomarkers for depression and other psychiatric disorders will be crucial for the successful advancement of diagnostics and targeted precision medicine. Ultra-high field MRI may provide additional pathophysiological insight for early disorder detection and effective intervention.

The VTA dopaminergic projections are the most well-established characterization of the “reward circuit”^18^. While there are no other 7 T functional MRI studies of VTA in humans with MDD, the current demonstration of VTA hyperconnectivity in MDD is in line with several pre-clinical models of depression. A well-validated rodent model of chronic social defeat stress is characterized by reduced social interaction and reduced sucrose preference, indicating an anhedonic and pro-depressive phenotype in susceptible animals^18–20^. This model is characterized by VTA hyperactivity as measured by electrophysiology, and normalizing VTA hyperconnectivity reduces the depressive, anhedonic phenotype^40^. Another recent preclinical study has demonstrated that VTA connectivity with ACC mediates motivation for rewards^41^ and heightened subgenual ACC connectivity has been previously demonstrated in patients with MDD^42^. Future studies should examine the functional organization of the broader cortical-subcortical reward circuit as a whole, which encompasses other neural substrates in the PFC, hippocampus and amygdala, as well as their roles in discerning and responding to environmental signals of reward, punishment and threat, in a larger sample size.

There are several general limitations of this work. Issues around patient contraindication, dizziness and claustrophobia are heightened at 7 T, meaning not all patients will be eligible to undergo scanning at this field strength. There also remain issues related to B0 and B1 inhomogeneity, resulting in distortion and drop-out, respectively, requiring advanced shimming and specialized pulse sequence designs^6^. The current study also had several specific limitations. Firstly, the voxel size was different between 7 T (2.5 mm^3^) compared to 3 T (3 mm^3^). Voxel size contributes to connectivity estimates, particularly for small nuclei such as the VTA^43^. Other ultra-high field MRI protocols have used smaller voxel sizes, one of which resulted in improved connectivity estimates in motor cortical regions, although this was not the case for other regions examined^43^. The precision of the whole-brain functional connectivity maps demonstrated in the current study may have been influenced by the smaller voxel size of the 7 T protocol compared to 3 T. While the voxel size at 7 T was smaller than 3 T, it was still relatively large compared to other studies^38,39^, a trade-off required for the use of the multi-echo acquisition protocol. The TR was also longer at 7 T compared to 3 T, meaning the number of frames was higher for 3 T for the same scan time. This is expected to enhance the temporal resolution of the 3 T scans compared to 7 T. The multiband (MB) factor was also higher at 3 T compared to 7 T. Higher MB factors allow faster scan times and better sampling of signal time courses for improved TSNR^44,45^ but can also result in signal leakage between slices and false positive activations^46^. MB factors lower than 9 at 3 T provide acceptable signal quality^46^ and since we report lower TSNR at 3 T compared to 7 T, we expect that these findings were not driven by false activations due to higher MB factor at 3 T. Finally, the iPAT acceleration factor was higher at 7 T compared to 3 T, which can reduce signal distortion, signal drop-out and partial volume effects but can also increase motion sensitivity and reduce SNR^5,47^. Multi-echo based acquisition and denoising strategies and higher channel head coils account for these issues in parallel imaging, by separating non-BOLD signals and reducing reconstruction errors, respectively. Therefore while we demonstrate improvements in multiple different metrics, including TSNR, connectivity metrics and whole-brain connectivity maps for both the same subjects and different subjects at 7 T compared to 3 T, some findings may be influenced by differences in acquisition protocols.

Another potential limitation of the current study is that the clinical characteristics of the MDD samples at 3 and 7 T where somewhat different, including a longer episode duration in the 3 T sample. We did not observe VTA hyper-connectivity in the 3 T sample so it is therefore conceivable that patients with longer duration may have attenuated connectivity. Finally, we examined normalized cross-correlation coefficients at 3 and 7 T for 470 regions, finding that although the networks were statistically similar, the similarity (*R* = 0.38) was lower than expected. This may be explained by individual differences in connectivity patterns, reflecting the limitation that the current analysis was not performed on entirely the same subjects. If the same subjects had been scanned on the same day, we would expect the similarity to be higher.

We provide a characterization of the utility of ultra-high field 7-T MRI for applications in functional connectivity mapping of neural structures that are critical to the pathophysiology of neuropsychiatric disorders. We emphasize the use of multi-echo based acquisition and denoising methods at ultra-high field.

## Supplementary information

Supplementary Information
