# Supplementary material for: Ultra-high field MRI reveals mood-related circuit disturbances in depression: a comparison between 3-Tesla and 7-Tesla
Source: Transl Psychiatry. 2019 Feb 15;9:94. doi: 10.1038/s41398-019-0425-6 (PMC6377652; doi:10.1038/s41398-019-0425-6)
Supplement: Supplementary file 1 — Supplementary Information [file 41398_2019_425_MOESM1_ESM.docx]

**Supplementary Information**

***Supplementary Table 1.*** *tSNR differences between 3T and 7T by region. Abbreviations: Ventral tegmental area (VTA), nucleus accumbens (Nac), dorsolateral prefrontal cortex (DLPFC), ventromedial prefrontal cortex (VMPFC), subgenual anterior cingulate cortex (SGACC), dorsal anterior cingulate cortex (DACC), T-statistic (t), p-value (p).*

|  | HC | MDD |
| --- | --- | --- |
| NAc | 21.65 | 59.60 |
| Amygdala | 13.87 | 101.02 |
| dlPFC | 62.55 | 106.24 |
| dACC | 37.20 | 74.93 |
| sgACC | 9.61 | 48.31 |
| vmPFC | 41.35 | -18.10 |

***Supplementary Table 2.*** *Improvement in ultra-high field 7-Tesla (7T) functional magnetic resonance imaging (MRI) cross-correlation coefficients for the ventral tegmental area (VTA) in subjects scanned with both 3-Tesla and 7T. Values indicate percent improvement in Fisher Z-normalised cross-correlation coefficients between the VTA and other regions 7T compared to 3T. Abbreviations: Nucleus accumbens (Nac), amygdala (Amyg), dorsolateral prefrontal cortex (DLPFC), ventromedial prefrontal cortex (VMPFC), subgenual anterior cingulate cortex (SGACC), dorsal anterior cingulate cortex (DACC).*

***Supplementary Figure 1.*** *Improved temporal signal to noise ratio (TSNR) at 7-Tesla (7T) compared to 3-Tesla (3T) in the same 5 healthy control subjects. Left: TSNR plotted for whole brain. Middle: TSNR plotted for individual echo times. Right: TSNR plotted for 7 regions of interest. Abbreviations: Echo time 1-4 (e1-e4), ventral tegmental area (VTA), nucleus accumbens (Nac), amygdala (Amyg), dorsolateral prefrontal cortex (DLPFC), ventromedial prefrontal cortex (VMPFC), subgenual anterior cingulate cortex (SGACC), dorsal anterior cingulate cortex (DACC).*

**

***Supplementary Figure 2.*** *Whole brain temporal signal to noise ratio (TSNR) plotted for healthy control (HC) and patients with major depressive disorder (MDD) at 3-Tesla (3T) and 7-Tesla (7T) functional magnetic resonance imaging.*

*
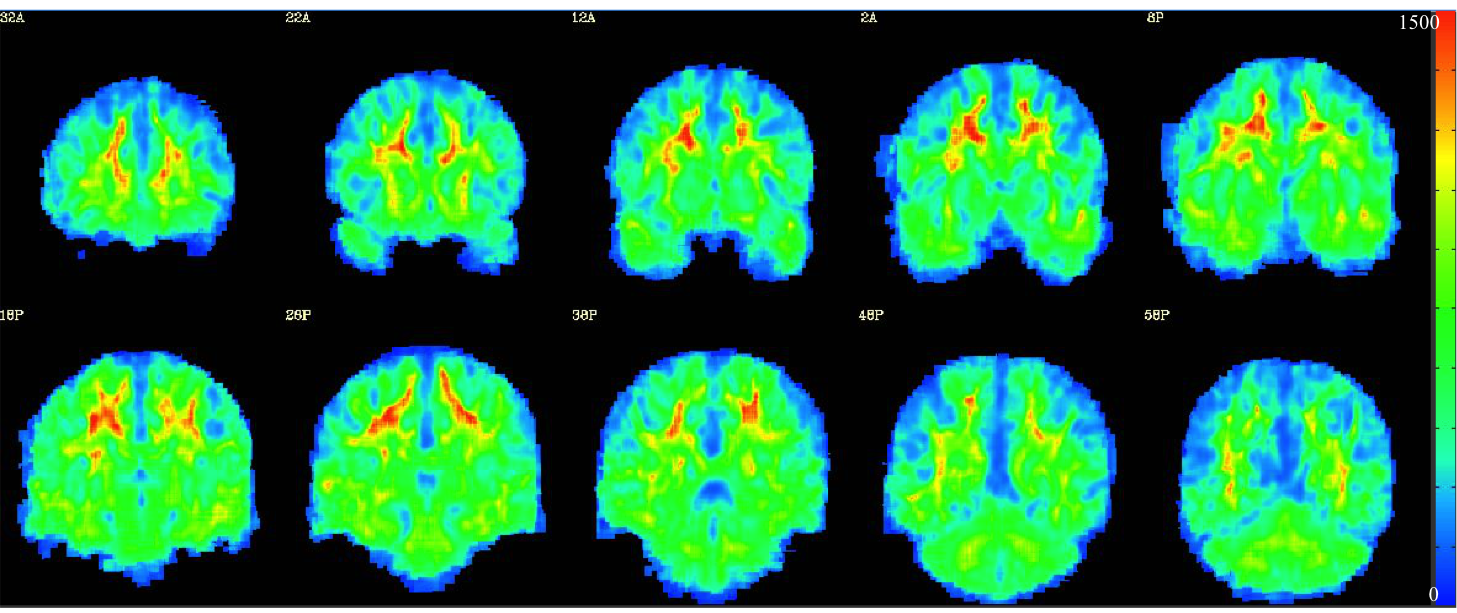
*

***Supplementary Figure 3.*** *Whole brain temporal signal to noise ratio (TSNR) map from 7-Tesla functional magnetic resonance imaging data.*
